# A Membrane Topology Model for Human Interferon Inducible Transmembrane Protein 1

**DOI:** 10.1371/journal.pone.0104341

**Published:** 2014-08-08

**Authors:** Stuart Weston, Stephanie Czieso, Ian J. White, Sarah E. Smith, Paul Kellam, Mark Marsh

**Affiliations:** 1 MRC Laboratory for Molecular Cell Biology, University College London, London, United Kingdom; 2 Wellcome Trust Sanger Institute, Wellcome Trust Genome Campus, Hinxton, United Kingdom; 3 MRC Centre for Medical Molecular Virology, Division of Infection and Immunity, University College London, London, United Kingdom; Institut Curie, France

## Abstract

InterFeron Inducible TransMembrane proteins 1–3 (IFITM1, IFITM2 and IFITM3) are a family of proteins capable of inhibiting the cellular entry of numerous human and animal viruses. IFITM1-3 are unique amongst the currently described viral restriction factors in their apparent ability to block viral entry. This restrictive property is dependant on the localisation of the proteins to plasma and endosomal membranes, which constitute the main portals of viral entry into cells. The topology of the IFITM proteins within cell membranes is an unresolved aspect of their biology. Here we present data from immunofluorescence microscopy, protease cleavage, biotin-labelling and immuno-electron microscopy assays, showing that human IFITM1 has a membrane topology in which the N-terminal domain resides in the cytoplasm, and the C-terminal domain is extracellular. Furthermore, we provide evidence that this topology is conserved for all of the human interferon-induced IFITM proteins. This model is consistent with that recently proposed for murine IFITM3, but differs from that proposed for murine IFITM1.

## Introduction

The InterFeron Inducible TransMembrane (IFITM) protein family of viral restriction factors was defined in 2009 in a screen for host-modifying proteins of influenza A virus (IAV) infection [1]. Initially identified over 30 years ago [2], and named 9–27 (IFITM1), 1-8D (IFITM2) and 1-8U (IFITM3) [3], these proteins received little attention until human IFITM3 depletion was found to enhance IAV infection in cell culture assays. Conversely, over-expression of IFITM3, or the closely related IFITM1 and IFITM2, could inhibit IAV replication [1]. Studies in mice and humans have suggested that IFITM3, at least, also protects against IAV infection *in vivo* [4], [5]. However, the IFITM proteins are not IAV-specific and, in cell culture at least, have broad-spectrum antiviral activity [6], though in some systems they may enhance cellular infection of human coronavirus OC43 [7] and human papillomavirus 16 [8].

Shortly after the identification of IFITM1-3 as antiviral factors, over-expression of IFITM3 was shown to cause an accumulation of intact IAV particles in endocytic organelles [9], suggesting that IFITM3 interfered with viral entry following endocytosis. Furthermore, this activity was dependent on localisation of IFITM3 to endosomes [10]. More recent studies suggested that IFITM over-expression may increase membrane rigidity and positive curvature, preventing the early events in membrane fusion [11]. Alternatively, IFITM protein interaction with vesicle membrane protein associated protein A (VAPA), disrupts cholesterol homeostasis and may increase membrane rigidity [12]. However, more recent work has questioned the role of cholesterol [13], [14]. Thus, the precise molecular mechanism(s) for IFITM inhibition of viral entry remains to be established.

**Figure 1:**
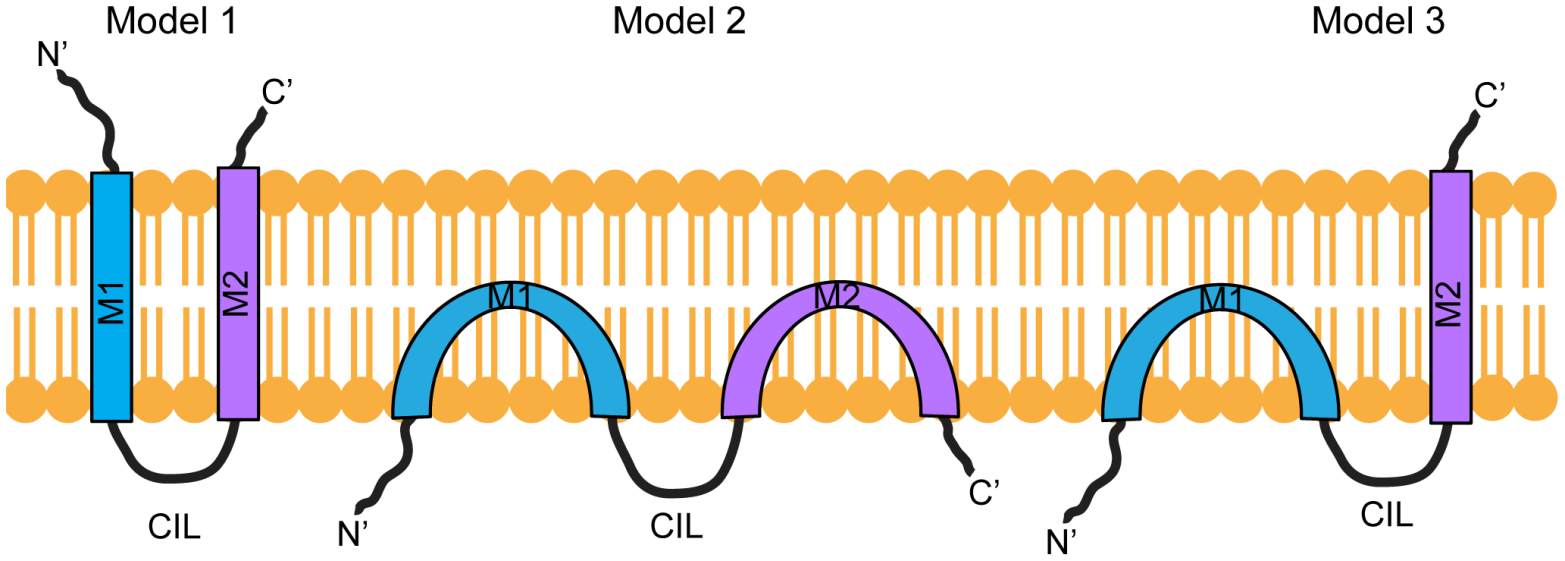
IFITM membrane topology models. In Model 1, the N- and C-terminal domains are extracellular and are connected by two transmembrane domains (M1 and M2) and the conserved intracellular loop (CIL). In Model 2 the two hydrophobic domains (M1, M2) do not span the membrane, resulting in NTD, CTD and CIL domain being located in the cytoplasm. In Model 3, the NTD and CIL domain are intracellular, suggesting M1 does not span the membrane, but the CTD is located on the extracellular side of the membrane and requires that M2 spans the membrane.

From their first descriptions, the IFITM proteins were thought to be membrane proteins [15]. Indeed, sequence analyses identified two hydrophobic, putative membrane interacting domains in each of the proteins. Additional studies demonstrated palmitoylation of cysteine residues adjacent to the hydrophobic domains, a post-translational modification indicative of membrane proteins [16]. However, the membrane topology of the IFITM proteins has remained ambiguous. Initially, they were suggested to be dual pass, transmembrane proteins with both N- and C-terminal domains (NTD and CTD) exposed extracellularly and a conserved intracellular loop (CIL) (Fig. 1, model 1). This model was based on the ability of antibodies against unknown IFITM1 external epitopes to aggregate leukaemia cells [15], [17], immunoprecipitation of extracellular radiolabelled IFITM1 [18] and the accessibility of IFITM3 NTD and CTD epitope tags at the cell surface by FACS and immunofluorescence assays, respectively [1].

This topology was subsequently challenged by studies demonstrating that the NTD and CIL domain of IFITM3 are post-translationally modified by cytoplasmic enzymes [10], [19]. Moreover, engineered myristoylation and prenylation sites at the NTD and CTD of murine IFITM3 can be detected as lipidated with selective chemical reporters alk-12 and alk-FOH, respectively [19]. These data suggested that both the NTD and CTD of IFITM3 are intracellular, and that the two hydrophobic sequences, enter, but do not span, the lipid bilayer [19] (Fig. 1, model 2). Murine IFITM1 was also proposed to have this topology [20].

Based on multiple approaches a third model has since been proposed for mu IFITM3, with the NTD and CIL facing the cytoplasm and the CTD located in the extracellular space, or within the lumen of vesicular organelles [21] (Fig. 1, model 3). While this manuscript was under revision, an additional study suggested a similar topology for human IFITM3 [22]. However, it remains unclear whether C-terminal epitope tags influence the topology, and whether the proposed topology applies to other human IFITM proteins.

Using immunofluorescence microscopy, protease cleavage assays, immuno-electron microscopy and biotin-labelling approaches, our data support the notion that the NTD of human IFITM1 is located in the cytoplasm, while the CTD is extracellular (Fig. 1, model 3). We provide evidence that the presence of a HA-tag at the IFITM1 C-terminus does not induce this topology. We also show that, although human IFITM2 and IFITM3 reside predominantly in intracellular membranes, they adopt the same topology. Together our data are consistent with the recently proposed model for mu IFITM3 [21], but not with that proposed for mu IFITM1 [20].

## Materials and Methods

### Cell lines and constructs

A549 (adenocarcinoma of human lung epithelia) cell lines stably expressing C-terminally HA-tagged human (hu) IFITM1, IFITM2 or IFITM3, as well as untransfected A549 cells [23], were cultured in Ham's F-12-GlutaMAX media (tissue culture reagents were from Life Technologies, unless indicated otherwise) supplemented with 10% (v/v) foetal calf serum (FCS) (PAA) and 1% (v/v) Penicillin/Streptomycin (Pen/Strep, 10,000 unit/ml/10,000 µg/ml). HEK293T cells were cultured in DMEM-GlutaMAX supplemented with 10% (v/v) FCS and 1% (v/v) Pen/Strep. All cell lines were maintained at 37°C and 5% CO_2_. Untagged IFITM1 and IFITM1-Vstop (a construct with a stop codon after Val109) were purchased from GeneArt and cloned into BamH1/Not1 sites of pcDNA3.1. Constructs were confirmed by sequencing (Source BioScience).

### Antibodies

Rat anti-HA (100 µg/ml, clone 3F10, Roche), mouse anti-HA (1 mg/ml, clone HA.11, 16B12 Covance), rabbit anti-IFITM1-NTD (100 µg/ml, Sigma), rabbit anti-IFITM3-NTD (250 µg/ml, Abgent), rabbit anti-VDAC (1 mg/ml, Abcam), rabbit anti-calreticulin (Thermo Scientific), mouse anti-tubulin (clone DM1A, ascites fluid, Sigma), goat anti-rat Alexa-488, goat anti-rabbit Alexa-488, goat anti-mouse Alexa-594 and goat anti-rabbit Alexa-647 (all 2 mg/ml, Life Technologies), goat anti-rabbit IRDye 680 and goat anti-mouse IRDye 800 (1 mg/ml, Li-COR), and wheat germ agglutinin (WGA) conjugated to Alexa-647 fluorophore (1 mg/ml, Invitrogen), were used at the dilutions given below.

### Permeabilised immunofluorescence

Cells cultured on coverslips were fixed in 3% (w/v) formaldehyde (FA) (TAAB) in phosphate buffered saline (PBS) for 15 minutes (min), quenched with 50 mM NH_4_Cl and 0.2% (w/v) bovine serum albumin (BSA, Sigma) in PBS (PBS/BSA) for 15 min and then permeabilised in PBS/BSA and 0.05% (w/v) saponin (Sigma) (permeabilisation buffer [PB]) for 30 min at room temperature (RT). Primary antibodies were diluted in PB and incubated with the coverslips for 1 hour (h). Unbound antibody was washed off with PB (3×5 min). Primary antibodies were detected with appropriate secondary antibodies conjugated to Alexa-488, Alexa-594 or Alexa-647, again, diluted in PB. Coverslips were mounted on Mowiol (Sigma) and imaged using a Leica TSC SPE confocal microscope.

### Intact cell immunofluorescence

Cells cultured on coverslips were washed with ice cold PBS/BSA then incubated with primary antibodies on ice for 1 h in ice cold PBS/BSA. Cells were washed to remove unbound antibody and fixed in ice cold 3% FA for 1 h (30 min on ice and 30 min at RT). Primary antibodies were detected with appropriate secondary antibodies in PBS/BSA. Cells were processed and imaged as described above.

### Wheat germ agglutinin staining

WGA-Alexa-647 was bound to cells on ice for 10 min. The cells were then washed four times with PBS/BSA and labelled using the intact cell staining procedure described above.

### Antibody feeding

Cells cultured on coverslips were incubated in media containing rat anti-HA for 3 h at 37°C. Unbound antibody was removed, and the cells then rinsed in PBS, fixed and permeabilised (as above) before incubation with anti-rat Alexa-488 in PB. Cells were mounted and imaged as described above.

For all immunofluorescence, the antibodies were used as follows: rat anti-HA 1∶100, rabbit anti-IFITM1-NTD 1∶200, rabbit anti-IFITM3-NTD 1∶200, mouse anti-tubulin 1∶100, goat anti-rat Alexa-488, goat anti-rabbit Alexa-488, goat anti-mouse Alexa-594 and goat anti-rabbit Alexa-647 all 1∶500 and WGA-Alexa-647 1∶200. Nuclei were stained with Hoechst-33258 (Sigma).

### Image analysis

To calculate the Pearson's R-value and Mander's correlation coefficients, M1 and M2, individual cells were segmented and analysed using the JACoP plugin on ImageJ software [24]. For M1 and M2 values, a Costes' automatic threshold was applied (as described [24]). To calculate the relative areas of yellow, red and green signals, images were initially split into the red and green component channels. These two images were then processed with the ‘AND’ function in ImageJ (producing an image of pixels that are only both red AND green). This image was subject to a manual threshold to observe only cellular structures and remove background noise. The pixel area was then calculated and these pixels defined as “yellow.” The “yellow” pixels were then super-imposed on the red and green single channel images, and removed from each of these (such that a pixel defined as “yellow” cannot be considered “red or “green”). The same approach of applying a threshold was then taken on the red channel to calculate the pixel area, with these being defined as “red.” Again, once calculated, these “red” pixels were super-imposed on the green channel image and removed. This allowed a threshold to be applied to the green channel and the area of the remaining pixels calculated. The relative area for each colour was calculated for each field of view and the mean average of all values calculated.

### qRT-PCR

The endogenous levels of *IFITM1*, *2*, and *3* mRNA in A549 and HEK293T cells were measured by QuantiTect SYBR green qRT-PCR (Qiagen) using the primers described in Table 1 and the following thermocycling conditions: RT step - 50°C for 30 min. PCR steps - 95°C for 15 min, 94°C for 15 s; 35 cycles of (94°C, 15 s; 60°C, 30 s; 72°C, 30 s) in a reaction volume of 50 µl.

**Table 1:** qRT-PCR primers.

Primer name	Sequence (5′ to 3′)
F'Human_IFITM3	ACTGTCCAAACCTTCTTCTCTC
R'Human_IFITM3	AGCACAGCCACCTCGTGCTC
F'Human_IFITM2	ATTGTGCAAACCTTCTCTCCTG
R'Human_IFITM2	ACCCCCAGCATAGCCACTTCCT
F'Human_IFITM1	AGCACCATCCTTCCAAGGTCC
R'Human_IFITM1	TAACAGGATGAATCCAATGGTC

A list of the primers used for qRT-PCR. F′ and R′ stand for forward and reverse, respectively.

Total RNA was extracted from a known number of cells (between 2.4×10^5^ and 5.9×10^5^) and quantitated (RNeasy minikit): 100 ng was used as a template in each qRT-PCR reaction.

Five standards from 10^7^–10^3^ copies were made using plasmids encoding the transcripts of human *IFITM1*, *2*, and *3*, using the following formula:
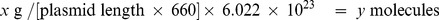



Using the standards for each transcript, the quantity of transcript was determined relative to the standard curve for 100 ng input RNA. The number of copies per cell was estimated by dividing the total number of cells by the total RNA extracted, multiplied by 100. This gave the equivalent number of cells that produced 100 ng of RNA and from this the RNA copy number per cell was inferred.

### Western blotting

Cells were lysed in Triton X-100 lysis buffer (1% [v/v] Triton X-100, 150 mM NaCl, 50 mM Tris-HCl pH 8.0 and 1× complete protease inhibitor cocktail [Roche]). Protein concentrations were determined using the BCA method (Thermo Scientific) following the manufacturer's instructions. Equal amounts of protein were mixed with reducing 3× Laemmli sample buffer (LSB), heated at 95°C for 5 min, separated by 15% SDS-PAGE and semi-dry transferred to a PVDF membrane (Immobilon-FL, Millipore). Membranes were blocked in TBST (Tris-buffered saline pH 7.4 [TBS] with 0.05% [v/v] Tween 20) containing 5% (w/v) dried skimmed milk (Marvel) for 1 h and incubated with primary antibodies at 4°C overnight. For trypsin cleavage assays, samples were collected (as described below) and lysed directly in 1× reducing LSB then heated at 95°C for 5 min. Equal volumes of cell lysates were loaded.

Antibodies used for protein detection were as follows: rabbit anti-IFITM3-NTD 1∶500, rabbit anti-IFITM1-NTD 1∶1000, mouse anti-HA 1∶1000 and rabbit anti-VDAC 1∶3000. The primary antibodies were detected using goat anti-rabbit IRDye 680 or goat anti-mouse IRDye 800 secondary antibodies, both at 1∶10,000 (in 5% milk-TBST), then imaged and quantified using an Odyssey system (Li-COR).

### Trypsin treatment

IFITM1-HA expressing A549 cells, grown in 6×35 mm well plates, were treated with 100 µg/ml trypsin (Sigma) for 5–30 min at 37°C. Subsequently, the cells were transferred to microcentrifuge tubes on ice, the wells were rinsed with 1 mg/ml soybean trypsin inhibitor (SBTI) (Sigma) in PBS, and the rinse added to the microcentrifuge tubes. Untreated control cells, as well as cells treated with inactivated trypsin (1∶1 volume ratio of SBTI to trypsin), were also collected. All samples were pelleted (5 min, 3000 RCF, 4°C) and washed with SBTI before direct lysis in LSB and western blotting, as described above.

### Flow cytometry

Cells in 35 mm dishes were treated with trypsin for 10 and 30 min or PBS for 30 min (as described above). The cells were then fixed, quenched (as previously) and washed with PBS/BSA prior to labelling with rat anti-HA antibody in PBS/BSA for 1 h at RT. The cells were then washed 3× with PBS/BSA and labelled with goat anti-rat Alexa-647 in PBS/BSA for 45 min. Cells were washed 3× with PBS and subject to flow cytometry (LSR-II; BD Bioscience). Cells were gated on forward and side scatter and analysed for fluorescence labelling. The data were processed using FlowJo vX.0.7 software (Tree Star). Antibodies were diluted as for immunofluorescence staining.

### Biotin labelling and pulldown

HEK293T cells, grown in 10 cm dishes, were transfected with untagged IFITM1 or IFITM1-Vstop expressing plasmids using FuGENE6. After 24 h, cells were detached with 5 mM EDTA and re-plated into 6×35 mm well plates. After a further 24 h, cells were labelled with 1 mg/ml EZ-link Sulfo-NHS-SS-Biotin (Thermo Scientific) for 45 min at 37°C. The biotin label was washed off with TBS and the cells lysed with 100 µl Triton X-100 lysis buffer (as previously). The whole cell lysate (70 µl) was added to a 25 µl NeutrAvidin agarose bead pellet (Thermo Scientific) and incubated for 3 h at 4°C on a rotor. Beads were then pelleted and the unbound material collected. The bead pellet was then washed with Triton X-100 lysis buffer, TBS and a TE buffer (10 mM Tris, 5 mM EDTA), prior to elution of proteins in 70 µl of 1× reducing LSB and heating at 95°C for 10 min. Eluate was collected and the elution procedure repeated. Equivalent volumes of whole cell lysate, unbound material and eluate from the NeutrAvidin beads were separated by 15% SDS-PAGE and western blotted as previously. Calreticulin was used as a loading control and detected with an anti-calreticulin antibody diluted 1∶5000 in 1% BSA-TBST.

### Electron microscopy

Cells were fixed with 4% (w/v) FA in 0.1 M phosphate buffer pH7.4, infused with 2.3 M sucrose, supported in 12% (w/v) gelatin and frozen in liquid nitrogen. Ultrathin (70 nm) cryosections were cut at −120°C and picked up in 1∶1 2% sucrose∶methylcellulose. Sections were labelled with primary antibody (mouse anti-HA 1∶400), followed by rabbit anti-mouse intermediate antibody (DAKO) and protein A gold, as described [25], [26]. Images were obtained using a Tecnai T12 transmission electron microscope (FEI) and captured using a Morada CCD camera (Olympus-SIS).

## Results

### The human IFITM1 C-terminal domain resides extracellularly

**Figure 2:**
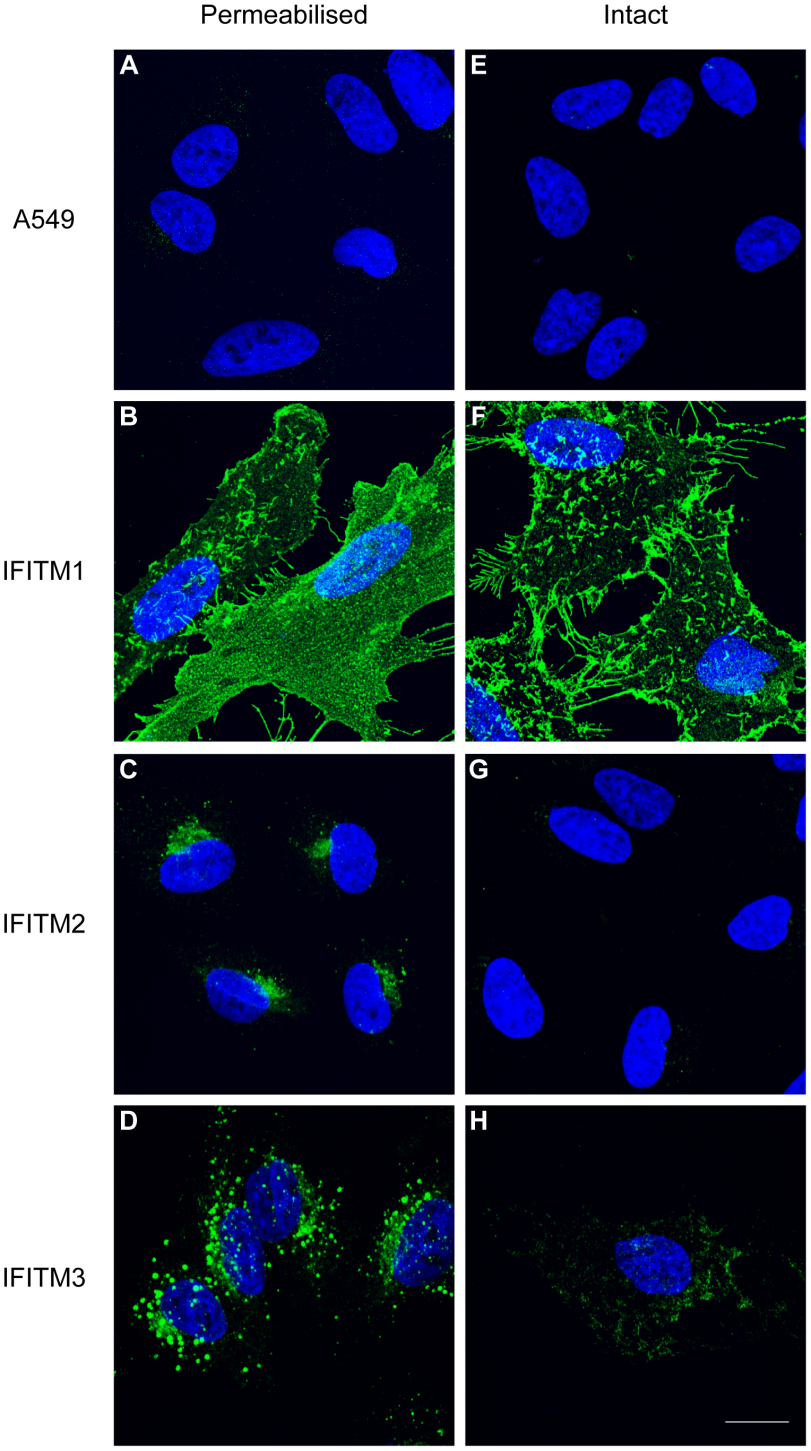
Cellular distribution of the human IFITM proteins. C-terminal domain HA-tagged IFITM1, IFITM2, IFITM3 and control A549 cell lines were stained intact, or following permeabilisation, with an anti-HA antibody and a secondary anti-rat Alexa-488 antibody. A) Permeabilised A549 cells show no specific staining. B-D) IFITM1-HA, IFITM2-HA and IFITM3-HA have distinct cellular distributions in permeabilised cells. E) Intact A549 cells (no detergent treatment) show no specific staining. F) Intact IFITM1-HA cells show positive cell surface HA staining. G) Intact IFITM2-HA cells show no detectable HA staining. H) Although the majority of intact IFITM3-HA cells show no anti-HA labelling, a minority (<1%) show low-level positive staining. Nuclei were labelled with Hoechst. All images are maximum projections of 0.25 µm optical sections taken through the depth of the cells using a confocal microscope. All images were taken using the same microscope settings and the levels adjusted uniformly. Scale bar represents 15 µm.

To investigate the cellular distributions of human (hu) IFITM1, 2 and 3 we used C-terminally HA-tagged proteins, stably expressed in A549 cells, and immunofluorescence microscopy. When cells were permeabilised and labelled with anti-HA antibodies, all three IFITM proteins were detected, and each had a different cellular distribution. IFITM1-HA was seen primarily at the plasma membrane (Fig. 2B). By contrast, IFITM2-HA and IFITM3-HA were seen mostly in intracellular compartments with distinct distributions. IFITM2-HA localised in a tight cluster of punctae close to the nucleus, while IFITM3-HA had a more dispersed, punctate distribution (Fig. 2C and D).

Cells were also stained in the absence of detergent, thus maintaining the integrity of the plasma membrane. For IFITM1, anti-HA labelling was very similar on intact and detergent-permeabilised cells (compare Fig. 2F and Fig. 2B). As a control for the integrity of the plasma membrane, cells were co-stained with an anti-tubulin antibody (Fig. S1). Detection of IFITM1-HA on non-permeabilised cells further demonstrated that the protein is located primarily at the cell surface in stable transfected A549 cells. Furthermore, the C-terminal HA-tag is accessible on intact cells and therefore located on the extracellular surface of the plasma membrane. Co-staining cells with fluorescent wheat germ agglutinin further confirmed this plasma membrane localisation (Fig. S2).

No anti-HA labelling was seen on intact IFITM2-HA expressing cells, in keeping with the notion that this protein is located on intracellular membranes (Fig. 2G). A similar result was seen for IFITM3-HA, although a low number of cells (<1%) did show some surface labelling with anti-HA antibodies (Fig. 2H and Fig. S3). This suggested that although the majority of IFITM3-HA is located on intracellular membranes (Fig. 2D), in some cells IFITM3-HA is either mis-sorted and/or is expressed at the cell surface where the C-terminal HA epitope is accessible.

### IFITM N-terminal domains reside intracellularly

The observation that the HA-tag of IFITM1 is accessible at the surface of intact cells prompted us to investigate the topology of the protein. We used two commercially available antibodies against the hu IFITM1 and IFITM3 NTDs. The IFITM1 NTD antibody (anti-IFITM1-NTD) was raised against a peptide encoding the first 35 amino acids of hu IFITM1. This domain has 69% and 75% amino acid identity with hu IFITM2 and IFITM3, respectively. The IFITM3 NTD antibody (anti-IFITM3-NTD) was raised against a peptide with a sequence corresponding to the first 30 amino acids of hu IFITM3. Hu IFITM1 has an N-terminal 21 amino acid truncation compared to IFITM3, so contains only 9 amino acids overlapping with this domain. However, there is 90% amino acid identity between the first 30 amino acids of hu IFITM3 and IFITM2.

**Figure 3:**
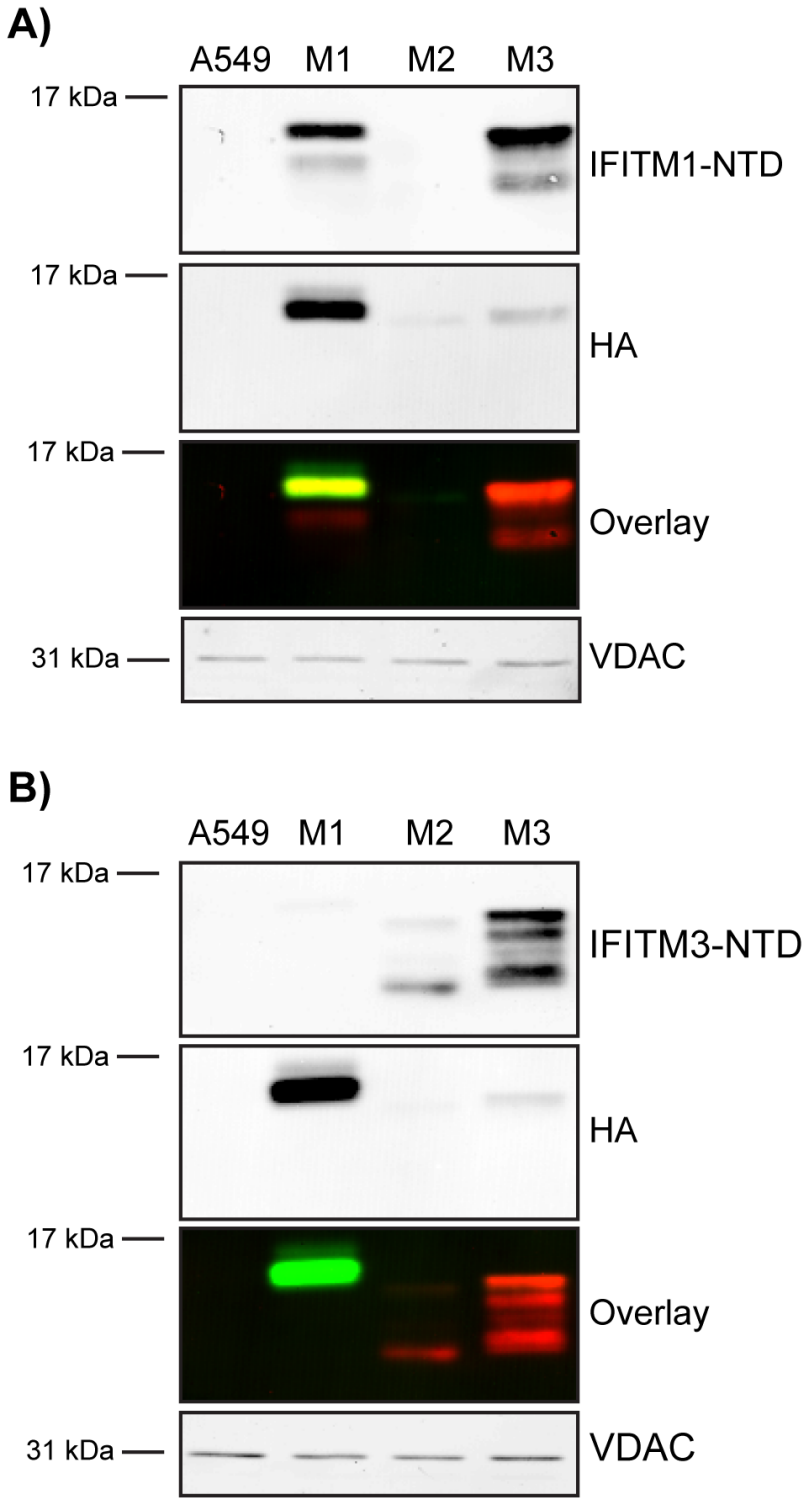
Analysis of IFITM NTD antibodies. Two commercially available antibodies targeting either the IFITM1-NTD or the IFITM3-NTD were screened by western blot to assess specificity using HA-tagged IFITM1-3 (M1, M2 and M3) cell lines along with control A549 cells. Proteins were also identified using the HA epitope. Blots were imaged on a Li-COR Odyssey system that uses far-red fluorophore conjugated secondary antibodies. In the overlay image, red represents anti-IFITM-NTD labelling and green represents anti-HA labelling. A) Anti-IFITM1-NTD detects IFITM1 and shows cross-reactivity with IFITM3. B) Anti-IFITM3-NTD detects IFITM3 and has cross-reactivity with IFITM2. VDAC was used as a loading control.

To determine the specificity of the NTD antibodies, both were tested on samples from the HA-tagged IFITM1-3 expressing A549 cells by western blot analysis. Anti-IFITM1-NTD detected IFITM1-HA and IFITM3-HA, but not IFITM2-HA (Fig. 3A). Anti-IFITM3-NTD detected both IFITM3-HA and IFITM2-HA, but not IFITM1-HA (Fig. 3B). No IFITM protein was detected in control A549 cells with either antibody by western blot. Multiple bands in the range of 12–17 kDa were seen with the NTD antibodies, but not the anti-HA, indicating possible post-translational modification of the proteins (see below).

**Figure 4:**
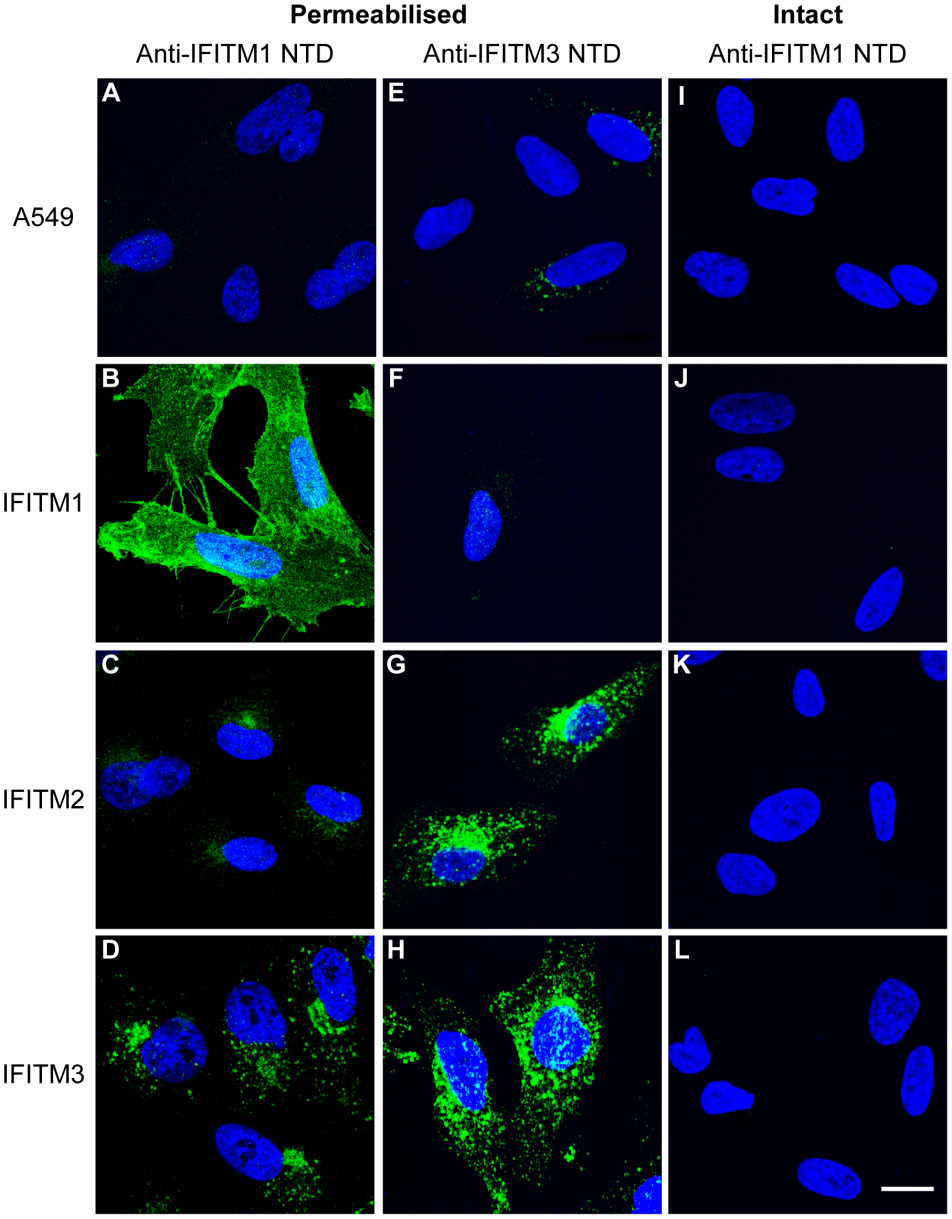
Analysis of the cellular distribution of the human IFITM proteins using anti-N-terminal domain antibodies. Immunofluorescence of A549 cells expressing HA-tagged IFITM1, IFITM2 and IFITM3 (or control A549 cells) using anti-IFITM1-NTD and anti-IFITM3 NTD antibodies. A) Permeabilised A549 cells show no specific staining. B-D) IFITM1-HA, IFITM2-HA and IFITM3-HA show distinct distributions in permeabilised cells. E and F) Anti-IFITM3-NTD detects low levels of endogenous protein in control A549 and IFITM1-HA cell lines. G and H) Anti-IFITM3-NTD detects IFITM2-HA and IFITM3-HA in permeabilised cells. I-L) No specific staining was seen on intact cells labelled with anti-IFITM1-NTD antibody. Nuclei were labelled with Hoechst. All images are maximum projections of 0.25 µm optical sections taken through the depth of the cells on a confocal microscope. All images were taken using the same microscope settings and the levels adjusted uniformly. Scale bar represents 15 µm.

When intact IFITM1, 2 or 3 cells were labelled with anti-IFITM1-NTD antibody, no staining was seen, suggesting intracellular NTDs (Fig. 4J, K and L). Following detergent treatment, all three IFITM expressing lines were labelled (Fig. 4B, C and D), though a weaker signal was seen for IFITM2, as expected from the western blot (Fig. 3A). By immunofluorescence, the anti-IFITM3-NTD antibody gave a low signal in the untransfected A549 control and IFITM1-HA cells (Fig. 4E and D), consistent with the low level of endogenous IFITM2 expression detected by qRT-PCR (Fig. S4). However, the antibody clearly detected IFITM2-HA and IFITM3-HA (Fig. 4G and H). The observed cellular distributions, using the anti-NTD antibodies, agree with those seen using anti-HA antibodies (Fig. 2), and the inaccessibility of the IFITM1-NTD to labelling on intact cells indicates the domain is on the cytoplasmic side of the plasma membrane.

### The IFITM1 C-terminal domain is accessible to extracellular proteases

**Figure 5:**
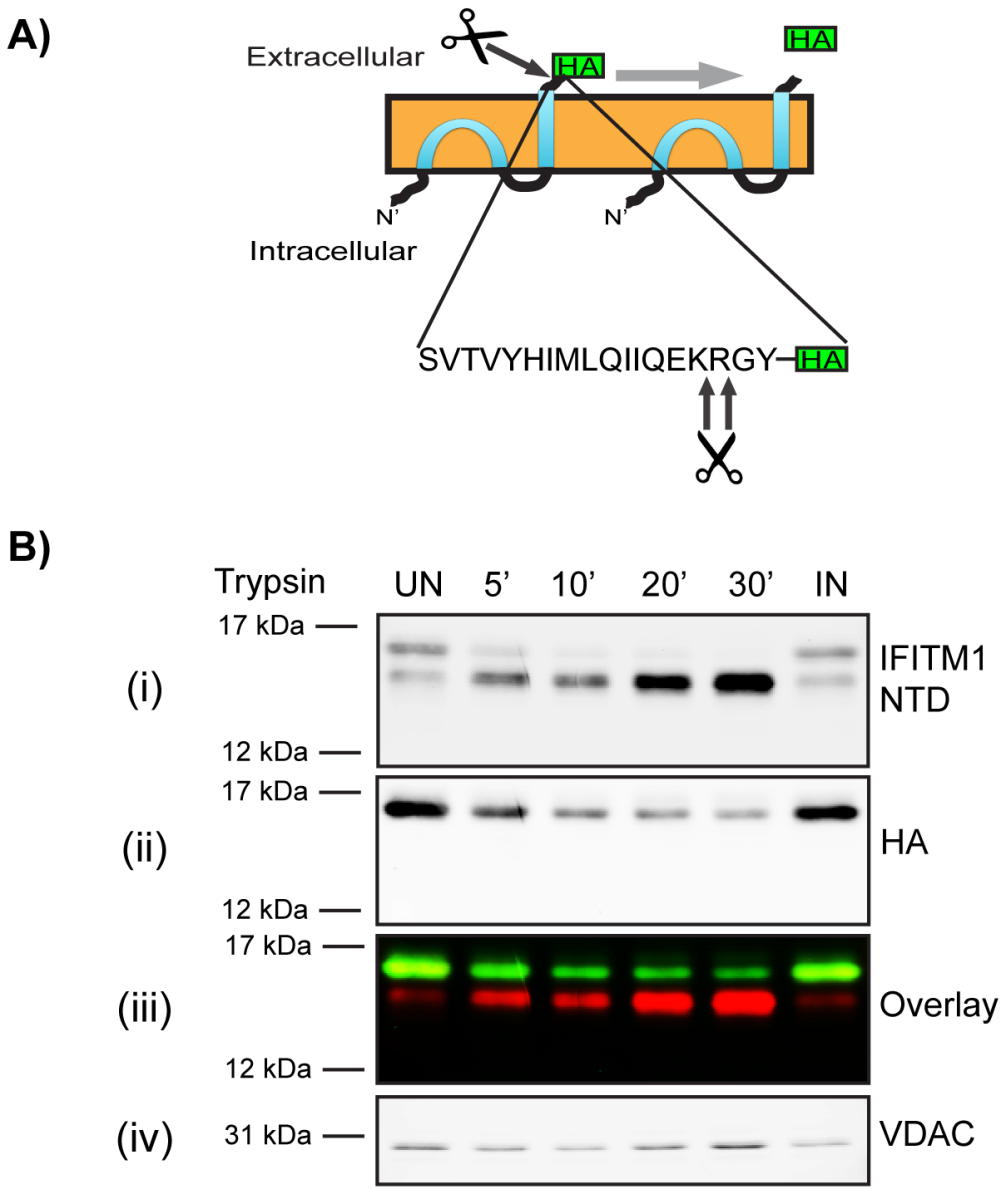
Trypsin cleavage of HA-tagged IFITM1. A) Predicted trypsin cleavage sites in hu IFITM1 CTD (Model 3, Fig. 1). B) IFITM1-HA cells were treated with exogenous trypsin for 5 to 30 mins at 37°C. The trypsin was inactivated with soybean trypsin inhibitor, the cells were lysed and the cellular proteins separated by SDS-PAGE. After transfer, proteins were identified with anti-IFITM1-NTD (i) and anti-HA (ii) antibodies. VDAC was used as a loading control (iii). Control samples were untreated (UN), or treated with SBTI-inactivated trypsin (IN). In the overlay image, red represents anti-IFITM1-NTD labelling and green represents anti-HA labelling (iv).

Immunofluorescence microscopy (Fig. 2 and 4) suggested that the IFITM1 C-terminal HA-tag resides on the extracellular face of the plasma membrane, while the NTD is cytoplasmic. To further investigate this topology, we used protease cleavage assays. Since IFITM1-HA is primarily localised to the plasma membrane we hypothesised that the CTD, and HA-tag, might be accessible to digestion by extracellular proteases. Following analysis of the human IFITM1 CTD sequence with the ExPASy ‘PeptideCutter’ tool (http://web.expasy.org/peptide_cutter/) we chose to use trypsin, which was predicted to cleave at two positions close to the C-terminus of hu IFITM1 and thus expected to release the HA-tag (Fig. 5A).

IFITM1-HA cells were incubated with trypsin for up to 30 min at 37°C. The trypsin was then inactivated by addition of excess soybean trypsin inhibitor (SBTI) and the cells analysed by western blot using antibodies against both the C-terminal HA-tag and the NTD. Analysis of samples from untreated cells with anti-IFITM1-NTD indicated two IFITM1 bands (Fig. 5Bi), as previously seen (Fig. 3A). In samples from trypsin-treated cells the higher molecular weight band was rapidly lost, with a concomitant increase in the intensity of the lower band (Fig. 5Bi). This suggested that the higher molecular weight band is IFITM1-HA (confirmed in an anti-HA and anti-IFITM1-NTD overlay analysis [Fig. 5Biii]), and that the lower band is a cleavage product that has lost the HA-tag. We noted that about 8% (relative to untreated samples) of the HA-tagged protein was not digested by trypsin, even after long periods, suggesting that a small pool of IFITM1-HA protein was inaccessible to the protease (see below).

We also analysed trypsin cleavage of the HA epitope by flow cytometry. Cells were treated with trypsin for 10 or 30 min and the trypsin inactivated with SBTI. Cells were then fixed and labelled with an anti-HA antibody and detected with an Alexa-647 conjugated secondary antibody. Untreated IFITM1-HA cells showed a high level of staining that was lost upon treatment with trypsin, as determined by a shift in the peak fluorescence intensity and decrease in mean fluorescence intensity (Fig. S5).

### Topology of untagged IFITM1

**Figure 6:**
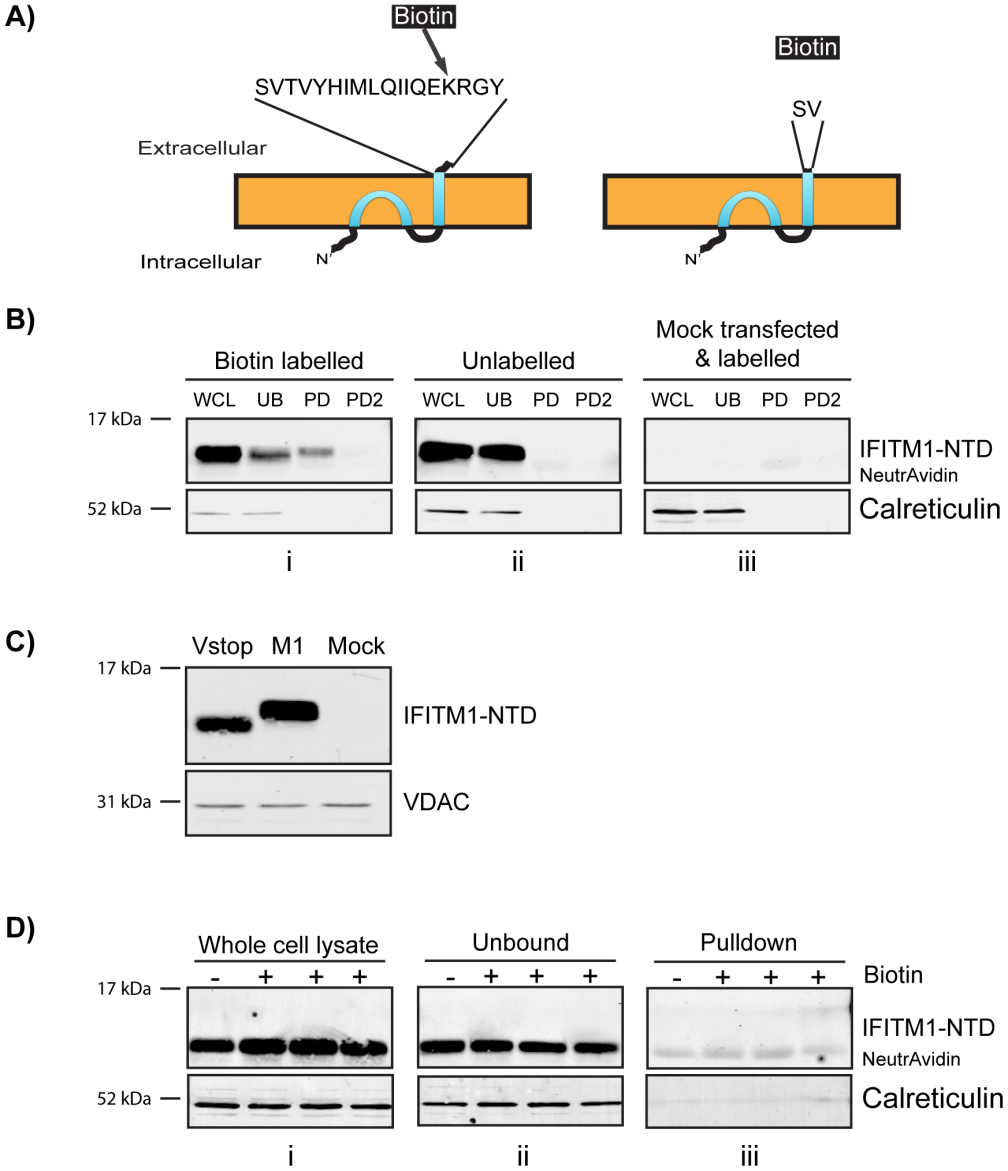
Biotin labelling and pulldown of untagged IFITM1. Untagged, wild type IFITM1 or IFITM1-Vstop expression plasmids were transfected into HEK293T cells. After two days the cells were labelled with cell impermeable Sulfo-NHS-SS-Biotin prior to incubation with NeutrAvidin agarose beads. A) Diagram to show the exposed CTD of IFITM1, with the targeted K122, or IFITM1-Vstop. B) Western blots probed with anti-IFITM1-NTD; WCL – whole cell lysates, UB – material that remained unbound by NeutrAvidin, PD1 and PD2 – two rounds of elution of protein from the NeutrAvidin beads. Gel i shows samples from cells labelled with biotin, gel ii shows unlabelled samples and gel iii shows samples from mock transfected HEK293T cells that were treated with Sulfo-NHS-SS-Biotin. NB. The elution step detached some NeutrAvidin monomers from the beads. These run at approximately 14 KDa and are seen as background bands in the western blots (labelled ‘NeutrAvidin’). Calreticulin was used as a loading control and negative control for pulldown specificity. C) Western blot comparing the wild type IFITM1 (M1) with IFITM1-Vstop (Vstop), along with mock transfected HEK293T cells. D) Western blots probed with anti-IFITM1-NTD for whole cell lysates (i) material that remained unbound to NeutrAvidin (ii) and protein eluted from the NeutrAvidin beads (iii). As previously, NeutrAvidin monomers were eluted, and have the same molecular weight at IFITM1-Vstop. This can be clearly seen in the pulldown blot due to the presence of a band in the unlabelled lane.

To exclude the possibility that C-terminal tagging influences the topology of IFITM1, we made use of a lysine residue (K122) present in the CTD that, if the observed topology is correct, will be accessible to labelling with NHS-biotin (Fig. 6A). To investigate this hypothesis, HEK293T cells were transfected with an untagged IFITM1 expression plasmid and incubated with cell impermeable Sulfo-NHS-SS-Biotin for 45 min at 37°C. While HEK293T cells appear to have a low level of IFITM1 mRNA (Fig. S4), no protein was detected by western blot of mock transfected cells (Fig. 6Biii).

Following biotin labelling of untagged IFITM1, cell lysates were precipitated using NeutrAvidin beads and analysed by western blot. IFITM1 labelled by biotin, and precipitated, is readily detected compared to unlabelled samples (Fig. 6B). The detection of IFITM1 in the biotin labelled, but unbound, fraction suggested that either the precipitation is not efficient, or only a proportion of IFITM1 has a CTD that is accessible to cell surface labelling.

To control for the specificity of labelling, a plasmid encoding an IFITM1 protein with a stop codon after the V109 codon (referred to as IFITM1-Vstop) that removes K122 (Fig. 6A), was transfected into HEK293T cells (Fig. 6C). After cells expressing this protein were incubated with Sulfo-NHS-SS-Biotin, none of the IFITM1-Vstop was absorbed to the beads (Fig. 6D).

### The IFITM1 CTD HA-tag resides on the outer leaflet of the plasma membrane

**Figure 7:**
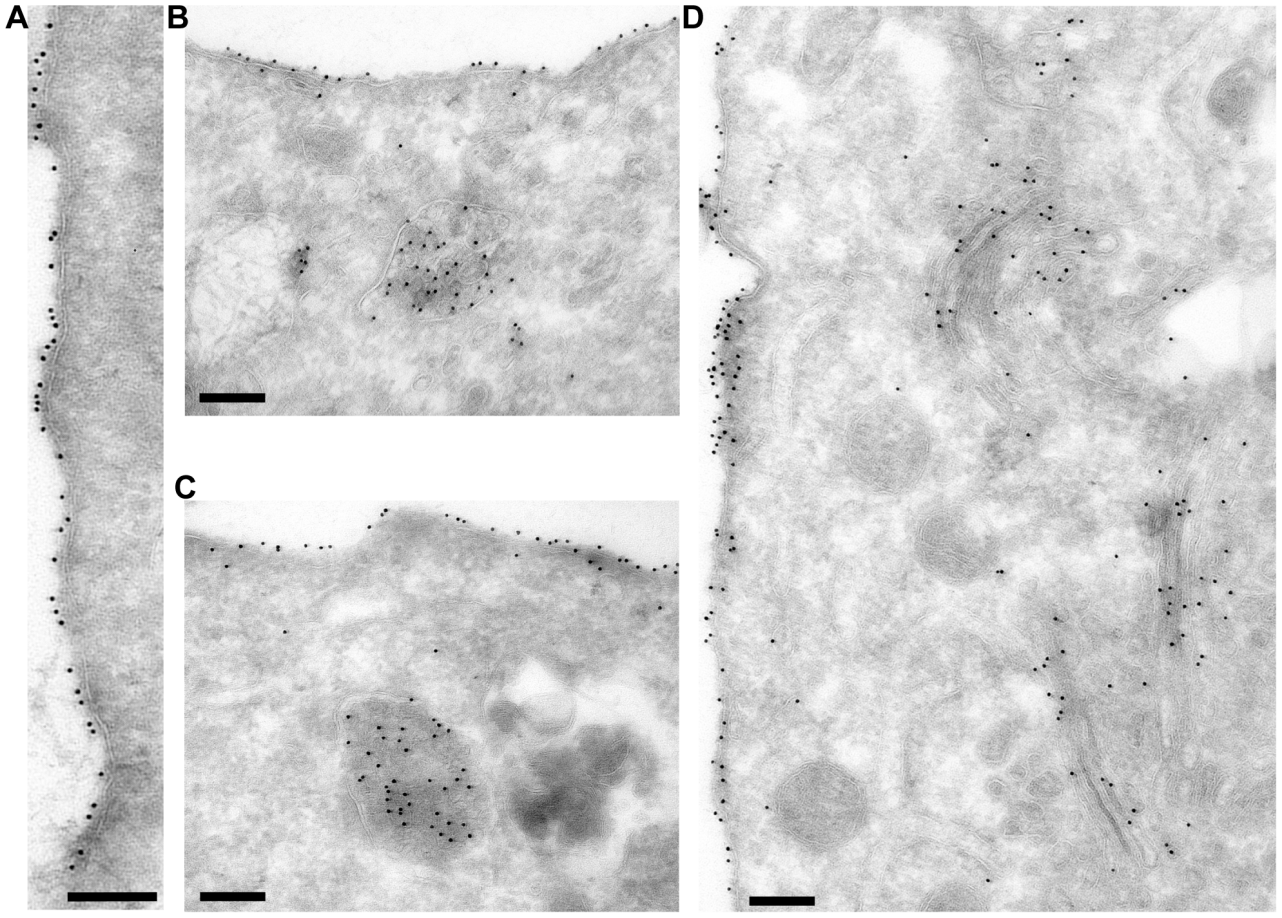
Immuno-gold labelling of A549 IFITM1-HA cell cryo-sections. Cryo-sections of the IFITM1-HA cells were labelled with anti-HA antibodies and Protein A gold. A) Plasma membrane labelling. B and C) Plasma membrane and multi-vesicular body labelling. D) Plasma membrane and Golgi apparatus labelling. Scale bars represent 200 nm.

To analyse hu IFITM1 in more detail, cryo-sections of IFITM1-HA cells were immuno-gold labelled for the HA-tag and examined by electron microscopy. As expected, labelling was seen predominantly at the plasma membrane (Fig. 7A and B). Close examination indicated that the majority of gold particles resided on the extracellular face of the plasma membrane, consistent with the notion that the hu IFITM1 CTD is located extracellularly. Labelling was also seen in the Golgi apparatus and in multi-vesicular bodies (Fig. 7C and D). These intracellular pools presumably indicate IFITM1 trafficking in the biosynthetic and endocytic pathways. This material would be inaccessible to extracellular proteases and labelling reagents, explaining the failure to completely digest IFITM1-HA with trypsin (Fig. 5B) and to fully label IFITM1 with biotin (Fig. 6B).

### Conservation of the observed hu IFITM1 topology in hu IFITM2 and IFITM3

Our results indicate that hu IFITM1 is located predominantly in the plasma membrane, making it highly amenable to the topological analysis described above. Conversely, we show that hu IFITM2-HA and IFITM3-HA are primarily intracellular and less accessible to these investigative methods. Alternative approaches were therefore adopted to determine the location of the HA-tagged CTD of hu IFITM2 or IFITM3.

**Figure 8:**
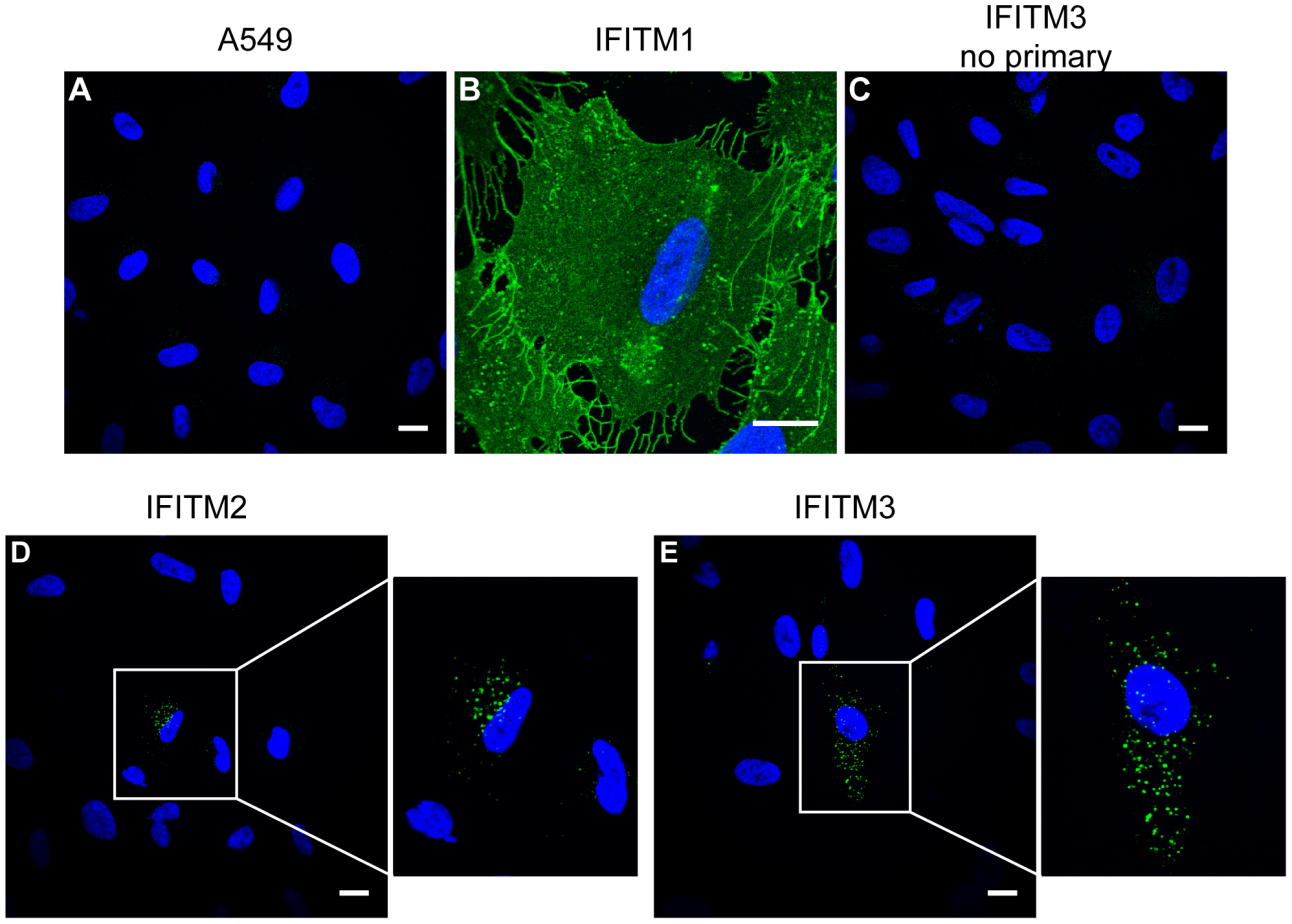
Antibody feeding of IFITM expressing cells. Live A549-IFITM-HA cells were incubated with 5 µg/ml anti-HA at 37°C to allow endocytosis of bound antibody molecules. Subsequently, the cells were washed, fixed, permeabilised and incubated with an anti-rat Alexa-488 conjugate. A) Control A549 cells show no specific staining. B) In IFITM1-HA cells the majority of labelling is at the plasma membrane. C) IFITM3-HA cells that were not incubated with anti-HA antibody show no labelling. For IFITM2-HA and IFITM3-HA expressing cells (D and E) the majority of cells are not labelled, however in both cases a minority of cells do show punctate labelling indicative of IFITM-mediated internalisation of anti-HA antibodies. In D and E, the boxed region has been enlarged. All images were taken using the same microscope settings and adjusted uniformly. Scale bars represent 15 µm.

An antibody-feeding approach was used in which live cells were cultured in medium containing anti-HA antibody for 3 h, prior to washing, fixation and visualisation by immunofluorescence microscopy. As expected, control untransfected A549 cells, incubated with anti-HA antibodies, and IFITM3-HA cells that were not incubated with anti-HA antibodies, showed no labelling (Fig. 8A and C). IFITM1-HA expressing cells acted as a positive control and showed strong cell surface labelling (Fig. 8B). By contrast, most IFITM2-HA and IFITM3-HA cells showed little, if any, labelling. However, approximately 25% of IFITM3-HA cells and 10% of IFITM2-HA cells (calculated from 30 random fields of view at 40X magnification) showed intracellular, punctate labelling (Fig. 8D and E). Since the cells were labelled prior to fixation, this intracellular labelling suggested that, in some cells, the IFITM2-HA and IFITM3-HA proteins are trafficked to the cell surface where an externally exposed HA epitope could bind antibody, prior to internalisation into intracellular organelles. The lack of labelling in the majority of cells, which express the IFITM proteins, indicates the labelling is not due to non-specific fluid phase uptake of antibody. Together, we conclude that, in a fraction of A549 cells, the CTDs of both IFITM2-HA and IFITM3-HA proteins are, at least transiently, exposed on the cell surface.

Previous results have suggested that IFITM2 and 3 are localised to endosomes (Fig. 2, 4 and [6]). The exposure of CTD HA-tags in the lumen of these organelles may result in their cleavage by endosomal/lysosomal proteases. This hypothesis is supported by western blots that showed low levels of HA-labelling for IFITM2 and 3 (Fig. 3). Moreover, when detected with anti-IFITM3-NTD, IFITM2-HA appears to have 3 bands in the range of 12–17 kDa, with the majority of the protein being of the lowest molecular weight, consistent with this protein having lost its HA-tag. A similar low molecular weight form was also seen for IFITM3-HA (Fig. 3).

IFITM expressing A549 cells were co-stained with antibodies against the NTD and the HA-tag. As IFITM proteins are relatively short (less than 133 amino acids) co-staining for the NTD and CTD should give apparent co-localisation. IFITM1-HA expressing cells showed a high degree of overlap between the anti-IFITM1-NTD and anti-HA antibodies (Fig. S6A). The overlap was seen across multiple images as demonstrated by Mander's correlation coefficients M1 and M2. Furthermore, analysis of the areas of different pixel colours demonstrated that around 70% (±1.8%) of pixels were detectable as yellow (Table S1).

**Figure 9:**
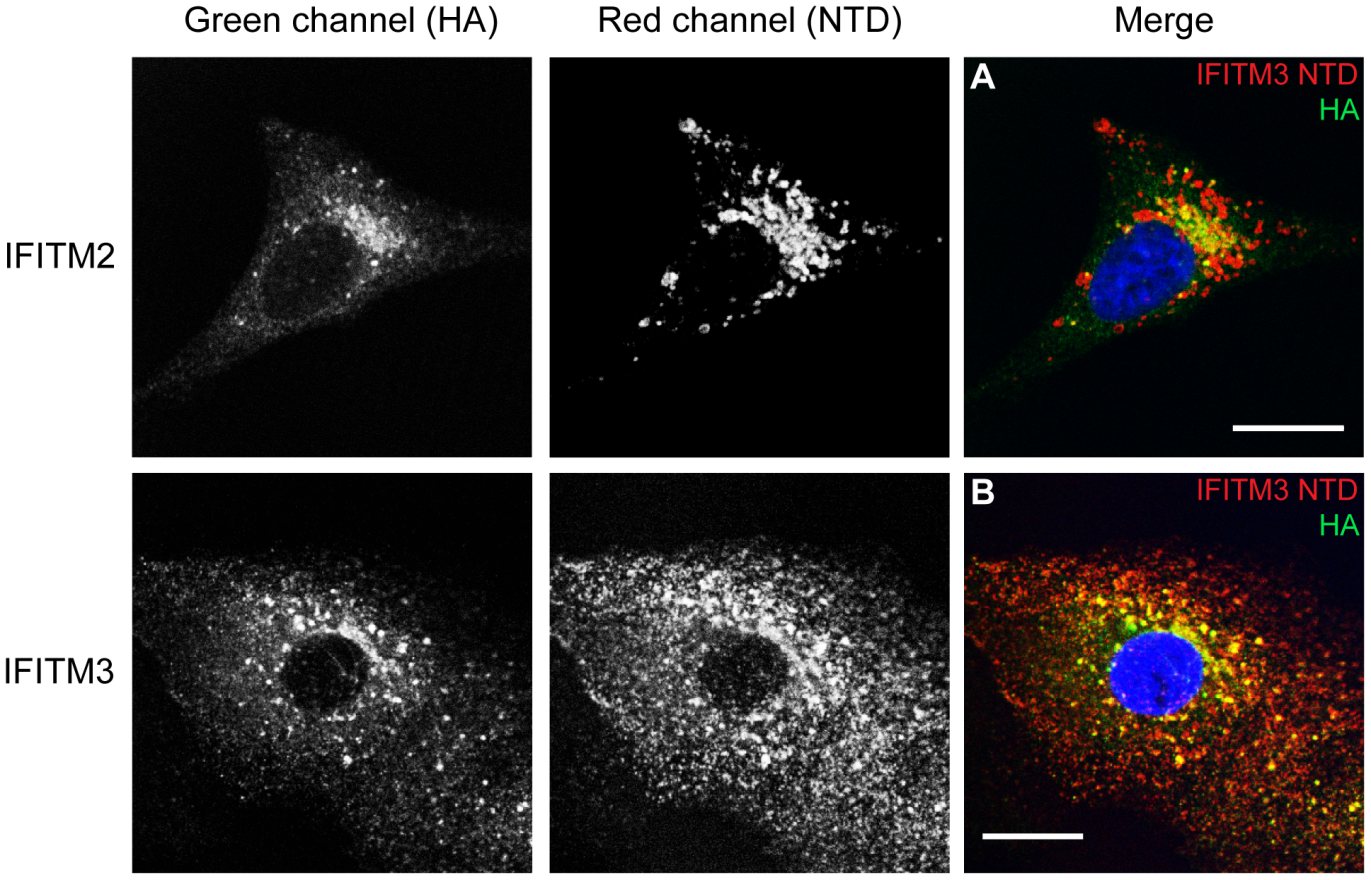
Co-staining with N- and C-terminal antibodies. Permeabilised IFITM2-HA (A) and IFITM3-HA (B) expressing cells were stained with antibodies against the C-terminal HA-tag (green [Alexa-488]) and the NTD, using the anti-IFITM3-NTD antibody (red [Alexa-647]). Images are single optical sections (0.25 µm thick) through the cells. Scale bars represent 15 µm. See also Table 2 and 3 for image analysis.

By contrast, on IFITM3-HA expressing cells, a lower level of co-localisation was seen with both NTD antibodies (Fig. 9B and Fig. S6C). Importantly, clear red punctae were visible, suggesting that in some organelles IFITM3 contains intact NTDs but lacks the CTD HA-tag. This conclusion is supported by the quantification of multiple images that demonstrate a lower Mander's M1 and M2, compared to IFITM1-HA, and show an excess of red pixels (55% [±1.4%]) for IFITM3-HA expressing cells (Table 2, 3 and Table S1).

**Table 2:** Image analysis of anti-IFITM3-NTD antibody and anti-HA antibody co-labelling.

Cell line	Number of cells imaged	Pearson's R value	Mander's M1	Mander's M2
IFITM2	57	0.73 (±0.13)	0.85 (±0.16)	0.86 (±0.14)
IFITM3	49	0.72 (±0.04)	0.75 (±0.21)	0.77 (±0.17)

Co-localisation analysis of multiple images, for each cell line, from two independent experiments. Pearson's R-value represents the correlation in intensity between the red (anti-IFITM3-NTD) and green (HA) channels. Mander's correlation coefficients, M1 and M2, represent the overlap of red, in pixels that are green, and the overlap of green, in pixels that are red, respectively. Error given is of the standard deviation.

**Table 3:** Image analysis of anti-IFITM3-NTD antibody and anti-HA antibody co-labelling.

Cell line	Fields of view	Yellow relative area	Red relative area	Green relative area
IFITM2	13	0.26 (±0.07)	0.49 (±0.11)	0.25 (±0.09)
IFITM3	14	0.27 (±0.04)	0.46 (±0.09)	0.27 (±0.09)

Relative areas of each colour were calculated as described in *materials and methods*. Red represents anti-IFITM3-NTD labelling, green represents anti-HA labelling and yellow represents overlap of the two stains. Error given is of the standard deviation.

The anti-IFITM1-NTD antibody does not detect IFITM2-HA as well as IFITM1-HA and IFITM3-HA (Fig. 3); we therefore used the anti-IFITM3-NTD antibody for a similar investigation of IFITM2-HA (Fig. 9A). When multiple images were quantified, the results for IFITM2-HA and IFITM3-HA expressing cells were largely equivalent, with 49% (±1.1%) of pixels being red for IFITM2-HA cells, compared to 46% (±0.9%) for IFITM3-HA cells (Table 2 and 3). The observed excess of red pixels for both IFITM2-HA and IFITM3-HA indicates loss of the HA-tag, consistent with the western blots (Fig. 3). Overall, the data suggest a topology in which the HA-tagged CTDs of IFITM2 and IFITM3 are exposed to the endosomal/lysosomal system.

## Discussion

The membrane topology of the interferon-induced IFITM proteins has been controversial. Initially it was suggested that both the NTD and CTD are located on the extracellular face of membranes and are connected by two transmembrane domains and the CIL domain (Fig. 1, model 1) [1], [15], [17], [18]. However subsequent studies, demonstrating post-translational modification of both the hu IFITM3 NTD and CIL domain by cytosolic enzymes, led to the proposal of a model that placed the NTD, CIL and CTD all within the cytoplasm (Fig. 1 model 2) [10], [19]. The same topology was also suggested for mu IFITM1 [20].

Here we present evidence that hu IFITM1, which localises primarily to the plasma membrane, has a topology with the NTD located in the cytoplasm and the CTD in the extracellular space (Fig. 1, model 3). Immunofluorescence microscopy showed that on non-permeabilised cells the NTD is not accessible to antibody labelling, whereas the CTD (through an HA-tag) is accessible. In agreement, the majority of tagged IFITM1 was accessible to cleavage by exogenous trypsin.

Our results also suggested that the HA-tag itself did not induce the extracellular CTD topology since untagged IFITM1 could be biotinylated at K122 using a cell impermeable biotin-labelling reagent. However, while a large proportion of untagged IFITM1 could be biotin labelled and absorbed to NeutrAvidin beads, some protein remained in the supernatant. We propose that the unlabelled protein is that which resides in intracellular compartments such as the Golgi and multi-vesicular bodies, as seen by EM (Fig. 7), and that there could also be accessibility issues at the plasma membrane due to the short length of the CTD.

Although not accessible on intact cells, the NTD of IFITM1 was accessible to labelling with antibodies following detergent treatment of cells and showed a very similar cellular distribution to that seen with CTD anti-HA labelling. The NTD contains two potential trypsin cleavage sites, but we saw no evidence of these sites being accessible to exogenous trypsin. Thus our results are consistent with a model recently proposed for murine and human IFITM3 indicating a cytoplasmic NTD and CIL domain, connected by an intramembrane hydrophobic domain (M1) that does not fully span the membrane, and a second transmembrane domain (M2) linking the CIL domain to an extracellular CTD [21], [22]. Our results with antibody labelling experiments indicate that hu IFITM2 and 3 are likely to have the same topology, suggesting this may be a common feature of human interferon-induced IFITM proteins, and mu IFITM3. However, this model is not consistent with published data on mu IFITM1 [20], in which palmitoylation of a CTD cysteine (mu IFITM1 C103) suggested a cytoplasmic location. This cysteine is not conserved in hu IFITM1, perhaps indicating why there is an apparent difference between hu and mu IFITM1 [20]. Whether palmitoylation of mu IFITM1 or other non-human IFITM proteins influence protein topology and/or its cellular distribution, as well as any possible effects on functional activities, remains to be examined.

The model for mu IFITM3 [21] and proposed here for hu IFITMs has important implications. Many studies of the IFITM proteins have used tagged constructs. If these tags are at the C-terminus they will reside in extracellular and lumenal spaces where they may be exposed to proteolytic conditions. Indeed, our results suggest that, even under normal culture conditions, IFITM-HA expressing cells have cleavage products resulting from the loss of the HA-tag or residues from the CTD itself. Western blots of A549 cells stably expressing IFITM1 show two bands when using the anti-IFITM1-NTD antibody. The higher molecular weight band is the HA-tagged form of the protein, while the lower weight band is a cleaved form of the protein that has lost the HA-tag, and possibly other residues from the CTD. Since this band is seen in cells that were not treated with trypsin, we suggest that this C-terminal cleavage may occur in the endosomal system. This conclusion is supported by the observation that in A549 cells expressing HA-tagged IFITM2 or 3, intracellular organelles can be seen that contain IFITM2 or 3 with intact NTD epitopes but lacking the CTD HA-tag (Fig. 9).

The potential for loss of the CTD or C-terminal tags will affect the interpretation of cell localisation studies that rely on CTD epitopes. IFITM3 has been localised to endosomal compartments that co-label with markers for early and late endosomes (transferrin, CD63, Rab proteins, LAMP1, LAMP2 and LysoTracker Red (as a marker for acidic compartments) [4], [9], [12], [27], [28]), but IFITM2 has proved harder to localise. Reliance on CTD epitope tags to determine the cellular distribution of the protein may not reveal the full cellular content of protein, and functionally important pools may be overlooked.

The relevance of the proposed topology to IFITM protein function is unclear at this stage. To date, published work has found that functionally important residues are located within the NTD [27] and the conserved CD225 domain [19], [29], comprising the membrane interacting domains and the CIL loop, as well as the second membrane domain that is proposed to interact with VAPA [12]. Our data suggest the first membrane domain enters into, but does not span, the lipid bilayers, perhaps allowing the protein to induce membrane curvature. It could be further speculated that an extracellular CTD may allow IFITM proteins to interact with key membrane components that are inaccessible on the cytoplasmic face of the membrane.

In conclusion, our data, together with those recently published for mu and hu IFITM3, provide a compelling case for hu IFITM proteins having an intracellular NTD and CIL domain, and an extracellular CTD. Whether all vertebrate IFITM proteins conform to the same organisation and, if not, the functional implications of other topologies, remain to be established.

## Supporting Information

Figure S1 **Tubulin co-staining on intact and permeabilised IFITM cell lines.** C-terminal domain HA-tagged IFITM1-3 and A549 cell lines were co-stained intact, or following permeabilisation, with an anti-HA antibody and an anti-tubulin antibody. These antibodies were detected with Alexa-488 (green) and Alexa-594 (red), respectively. A) Permeabilised IFITM-HA cells show positive labelling for all IFITM proteins and tubulin. B) Intact IFITM-HA cells show no labelling for tubulin, indicating that the plasma membrane has remained intact and the antibody does not have access to the cytoplasm. As previously, IFITM1-HA can be labelled on intact cells, IFITM2-HA has no labelling and only a minority of IFITM3-HA expressing cells shows plasma membrane labelling. Nuclei were labelled with Hoechst. All images are maximum projections of 0.25 µm optical sections taken through the depth of the cells on a confocal microscope. All images were taken using the same microscope settings and the levels adjusted uniformly. Scale bars represent 15 µm.
(TIF)

Figure S2 **Wheat germ agglutinin co-staining on intact IFITM cell lines.** IFITM-HA cell lines co-labelled with anti-HA antibody, detected with Alexa-488 (green channel) and WGA-Alexa-647 (red channel). Images are of a single optical section (0.25 µm thick) through the middle surface of the cells. Scale bars represent 15 µm.
(TIF)

Figure S3 **Immunofluorescence of intact IFITM3 cells.** Intact IFITM3-HA cells stained with anti-HA antibody. A minority (<1%) of the cells show some plasma membrane labelling, although the vast majority do not. Labelling of permeabilised cells showed that all cells express IFITM3-HA (Fig. 2D) Scale bar represents 15 µm. The boxed region is enlarged in the right hand panel.
(TIF)

Figure S4 **qRT-PCR of A549 and HEK293T cells.** qRT-PCR of A549 and HEK293T cells to determine the expression levels of any endogenous IFITM proteins. Each bar is labelled with the mean number of RNA copies per cell with error bars representing the standard deviation from n = 3 amplifications.
(TIF)

Figure S5 **Trypsin cleavage and flow cytometry analysis of IFITM1-HA.** IFITM1-HA cells were treated with exogenous trypsin for 10 and 30 mins at 37°C. The trypsin was inactivated with soybean trypsin inhibitor, and cells fixed then labelled with anti-HA antibody. The HA labelling was detected with anti-rat Alexa-647 and the cells analysed by flow cytometry. A) Histograms representing the fluorescence intensity of HA labelling. The black line represents control A549 cells expressing no HA constructs. The green line represents untreated IFITM1-HA cells. The blue and red lines represent 10 and 30 mins of trypsin treatment, respectively. B) Mean fluorescence intensity of HA labelling. Data represent mean averages from n = 2 cleavages and error bars equal standard deviation.
(TIF)

Figure S6 **Co-staining with anti-IFITM1-NTD and anti-HA antibodies.** Permeabilised IFITM1-HA (A), IFITM2-HA (B) and IFITM3-HA (C) expressing cells were stained with antibodies against the C-terminal HA-tag (green [Alexa-448]) and the NTD, using the anti-IFITM1-NTD antibody (red [Alexa-647]). Images are of single optical sections (0.25 µm thick) through the middle the cell. Scale bars represent 15 µm.
(TIF)

Table S1 **Image analysis of anti-IFITM1-NTD antibody and anti-HA antibody co-labelling.** Co-localisation analysis of multiple images, for each cell line, from three independent experiments. Pearson's R-value represents the correlation in intensity between the red (anti-IFITM1-NTD) and green (HA) channels. Mander's correlation coefficients, M1 and M2, represent the overlap of red, in pixels that are green, and the overlap of green, in pixels that are red, respectively. Relative areas of each colour were calculated as described in *materials and methods*. Error given is of the standard deviation.
(DOCX)
