# Supplementary figures and images for: A Membrane Topology Model for Human Interferon Inducible Transmembrane Protein 1
Source: PLoS One. 2014 Aug 8;9(8):e104341. doi: 10.1371/journal.pone.0104341 (PMC4126714; doi:10.1371/journal.pone.0104341)

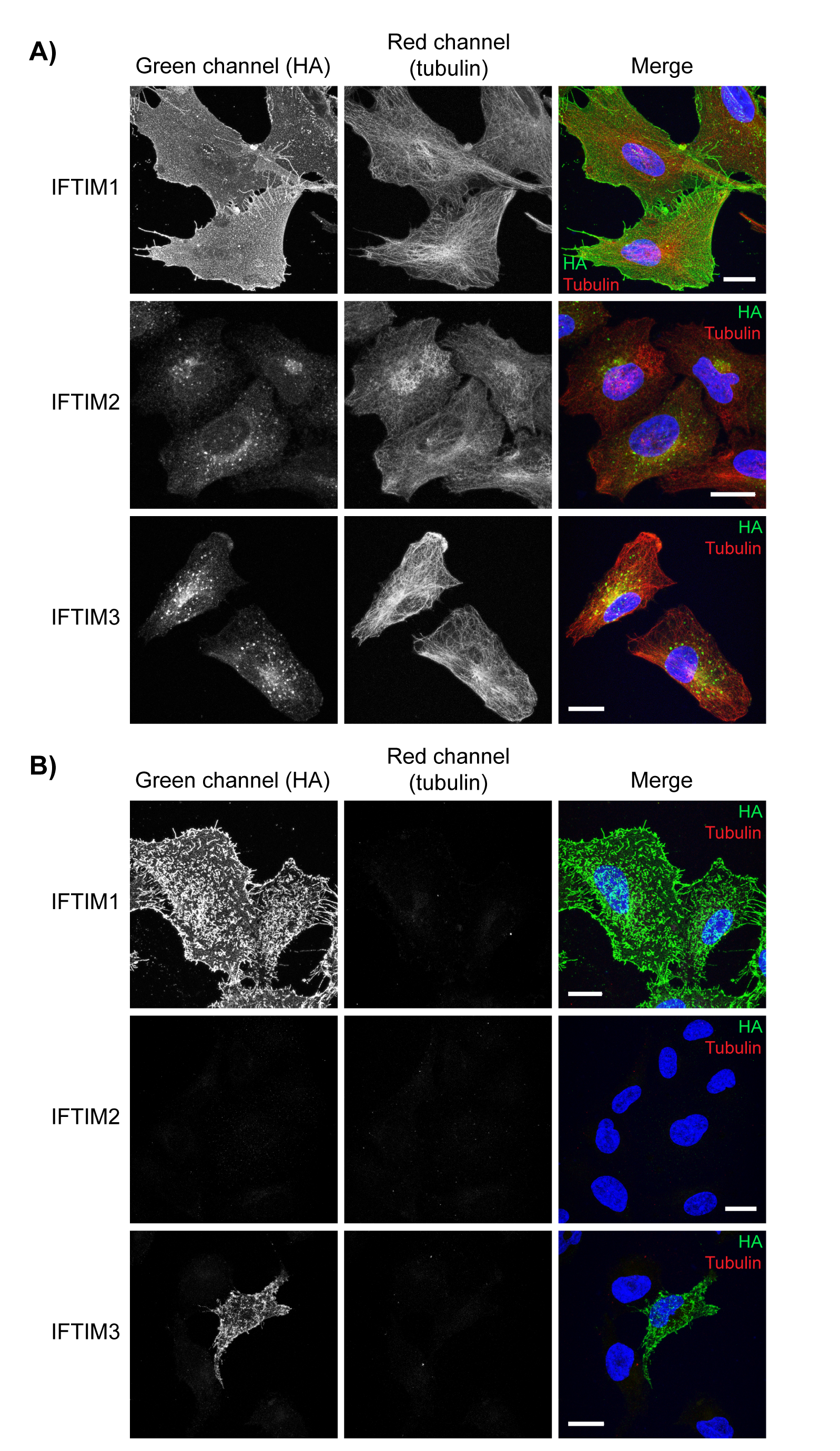

Supplement: Figure S1 — Tubulin co-staining on intact and permeabilised IFITM cell lines. C-terminal domain HA-tagged IFITM1-3 and A549 cell lines were co-stained intact, or following permeabilisation, with an anti-HA antibody and an anti-tubulin antibody. These antibodies were detected with Alexa-488 (green) and Alexa-594 (red), respectively. A) Permeabilised IFITM-HA cells show positive labelling for all IFITM proteins and tubulin. B) Intact IFITM-HA cells show no labelling for tubulin, indicating that the plasma membrane has remained intact and the antibody does not have access to the cytoplasm. As previously, IFITM1-HA can be labelled on intact cells, IFITM2-HA has no labelling and only a minority of IFITM3-HA expressing cells shows plasma membrane labelling. Nuclei were labelled with Hoechst. All images are maximum projections of 0.25 µm optical sections taken through the depth of the cells on a confocal microscope. All images were taken using the same microscope settings and the levels adjusted uniformly. Scale bars represent 15 µm. (TIF) [file pone.0104341.s001.tif]

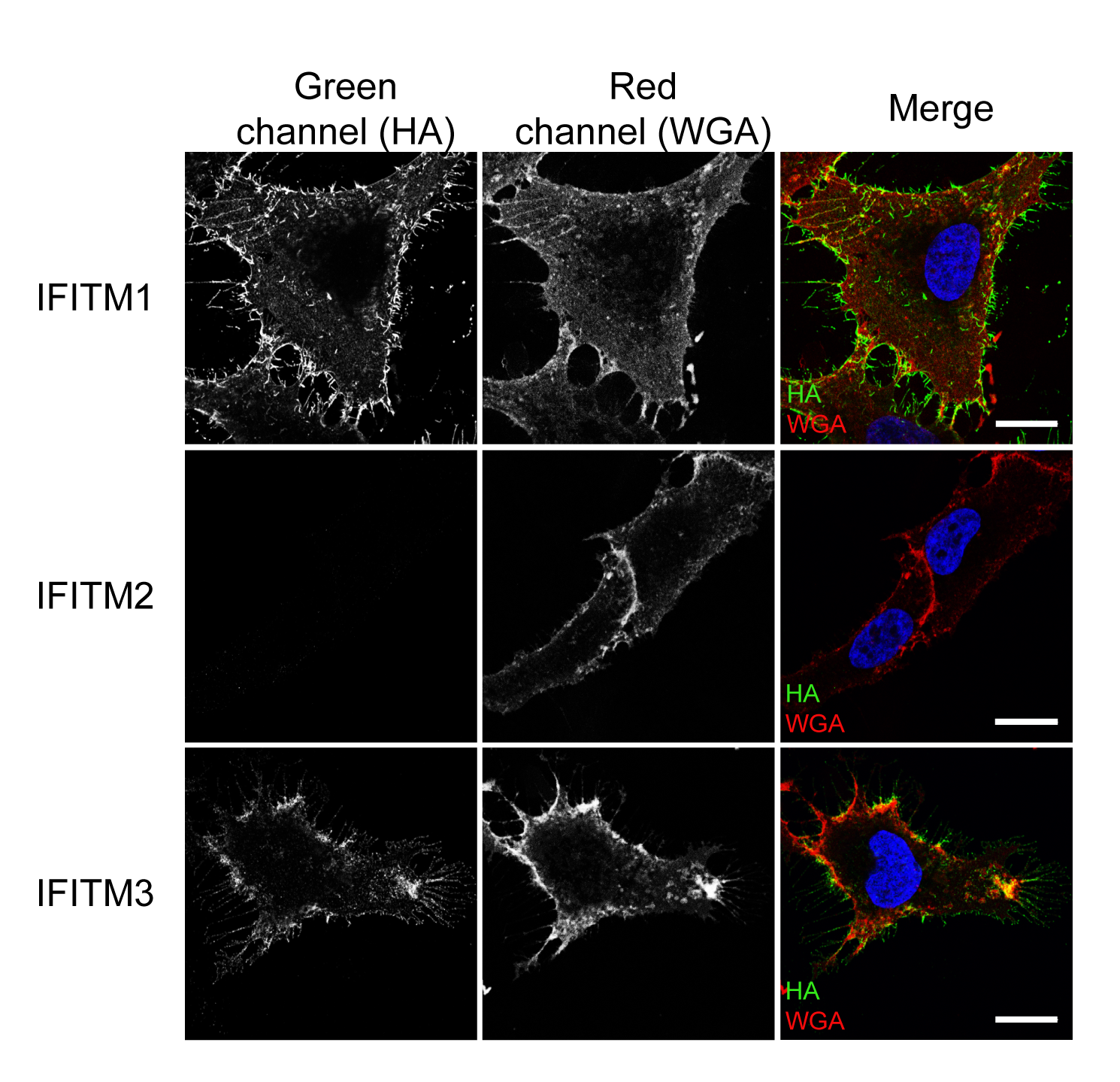

Supplement: Figure S2 — Wheat germ agglutinin co-staining on intact IFITM cell lines. IFITM-HA cell lines co-labelled with anti-HA antibody, detected with Alexa-488 (green channel) and WGA-Alexa-647 (red channel). Images are of a single optical section (0.25 µm thick) through the middle surface of the cells. Scale bars represent 15 µm. (TIF) [file pone.0104341.s002.tif]

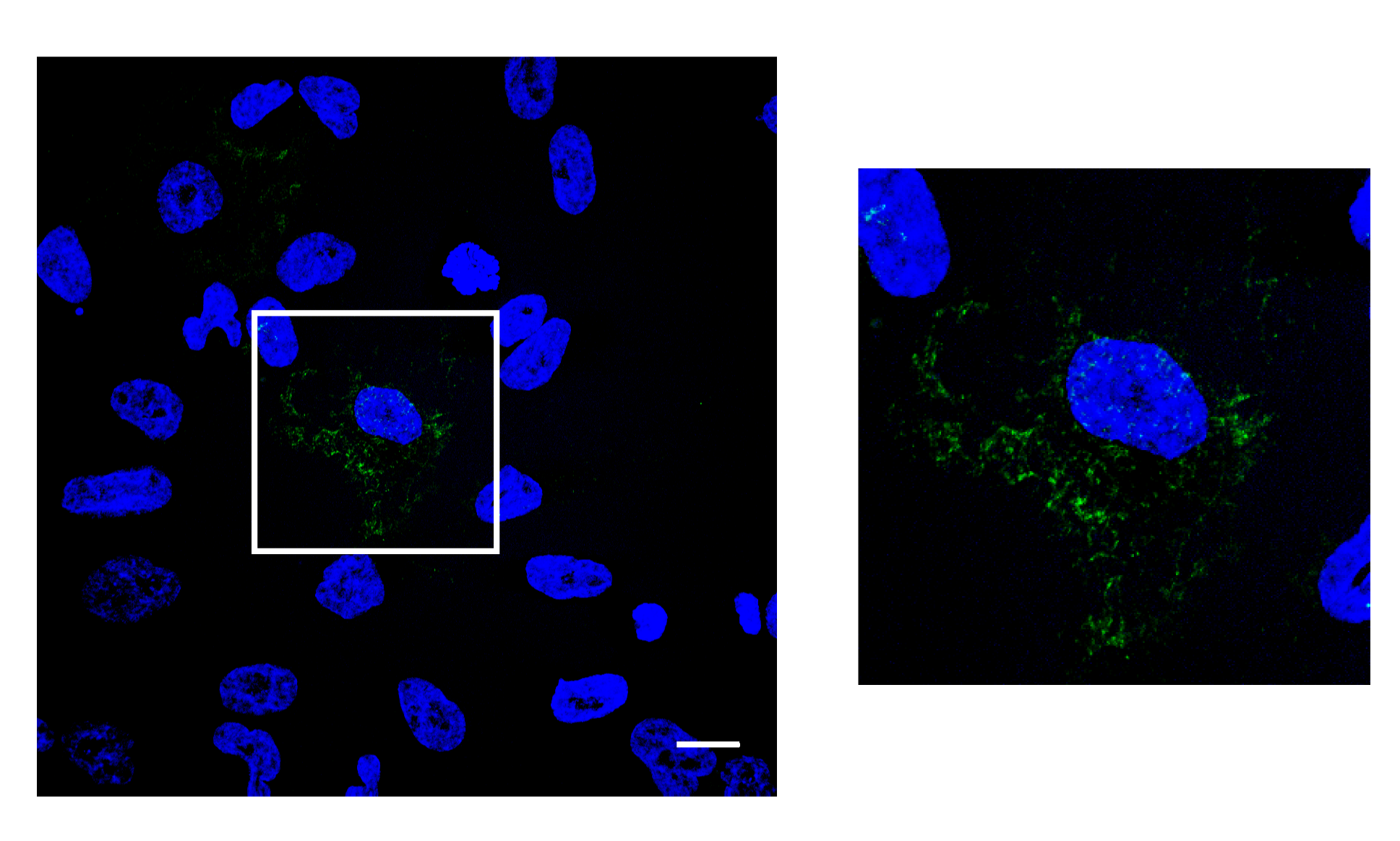

Supplement: Figure S3 — Immunofluorescence of intact IFITM3 cells. Intact IFITM3-HA cells stained with anti-HA antibody. A minority (<1%) of the cells show some plasma membrane labelling, although the vast majority do not. Labelling of permeabilised cells showed that all cells express IFITM3-HA (Fig. 2D) Scale bar represents 15 µm. The boxed region is enlarged in the right hand panel. (TIF) [file pone.0104341.s003.tif]

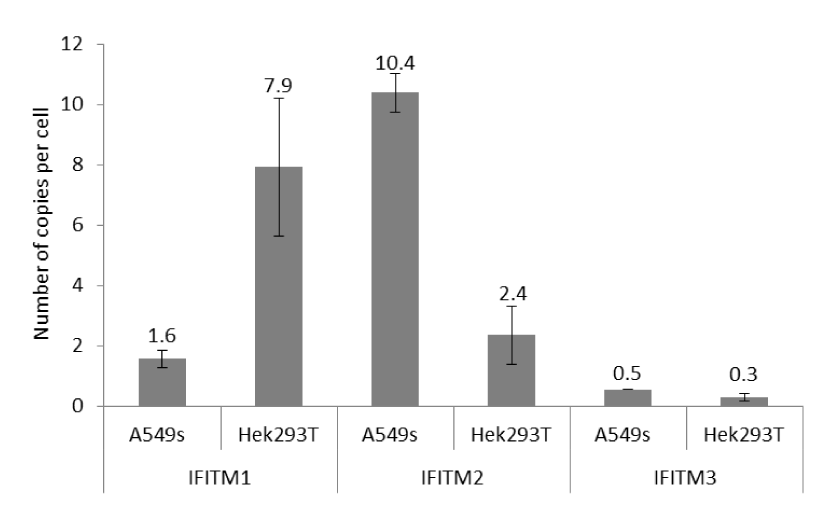

Supplement: Figure S4 — qRT-PCR of A549 and HEK293T cells. qRT-PCR of A549 and HEK293T cells to determine the expression levels of any endogenous IFITM proteins. Each bar is labelled with the mean number of RNA copies per cell with error bars representing the standard deviation from n = 3 amplifications. (TIF) [file pone.0104341.s004.tif]

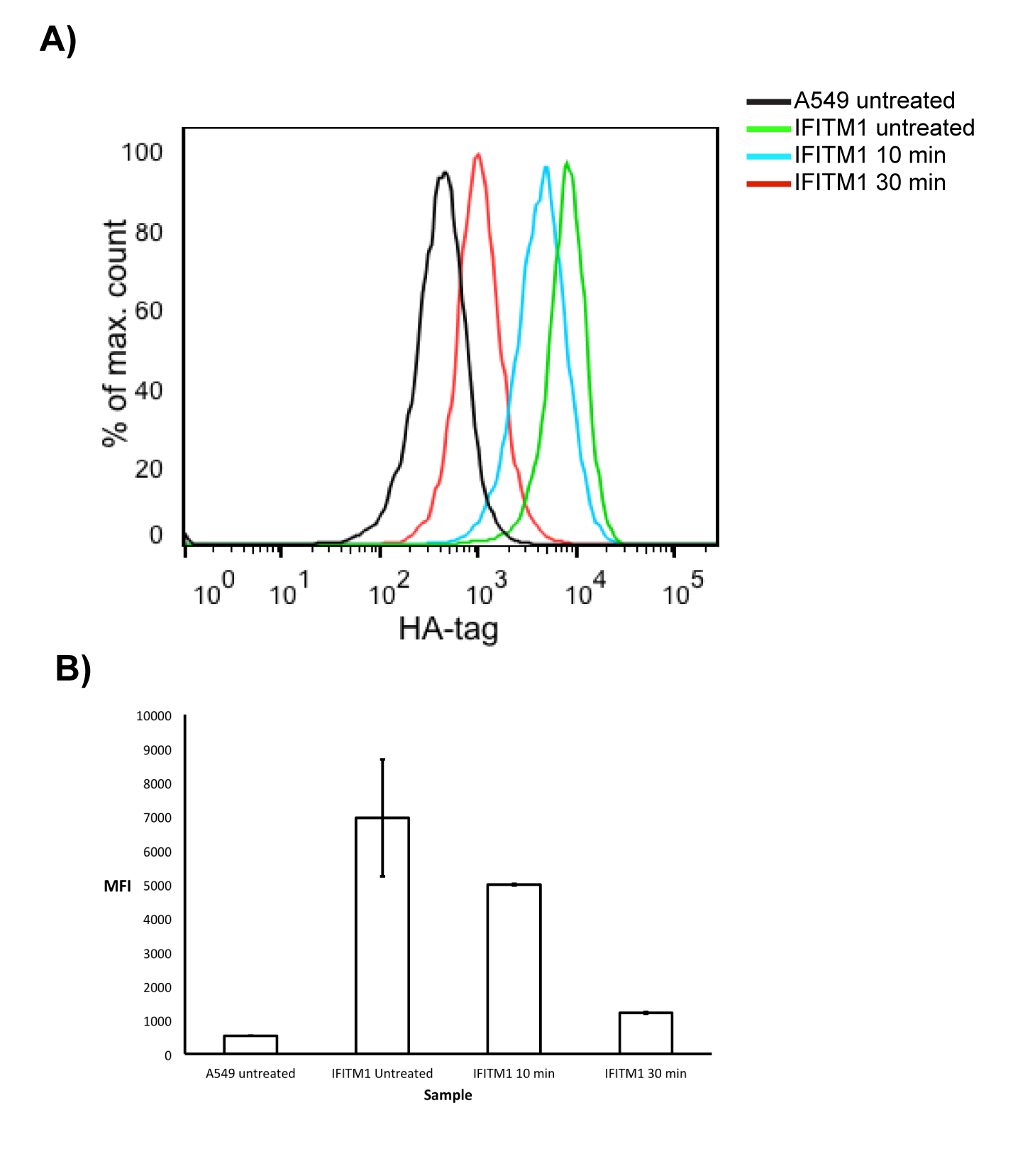

Supplement: Figure S5 — Trypsin cleavage and flow cytometry analysis of IFITM1-HA. IFITM1-HA cells were treated with exogenous trypsin for 10 and 30 mins at 37°C. The trypsin was inactivated with soybean trypsin inhibitor, and cells fixed then labelled with anti-HA antibody. The HA labelling was detected with anti-rat Alexa-647 and the cells analysed by flow cytometry. A) Histograms representing the fluorescence intensity of HA labelling. The black line represents control A549 cells expressing no HA constructs. The green line represents untreated IFITM1-HA cells. The blue and red lines represent 10 and 30 mins of trypsin treatment, respectively. B) Mean fluorescence intensity of HA labelling. Data represent mean averages from n = 2 cleavages and error bars equal standard deviation. (TIF) [file pone.0104341.s005.tif]

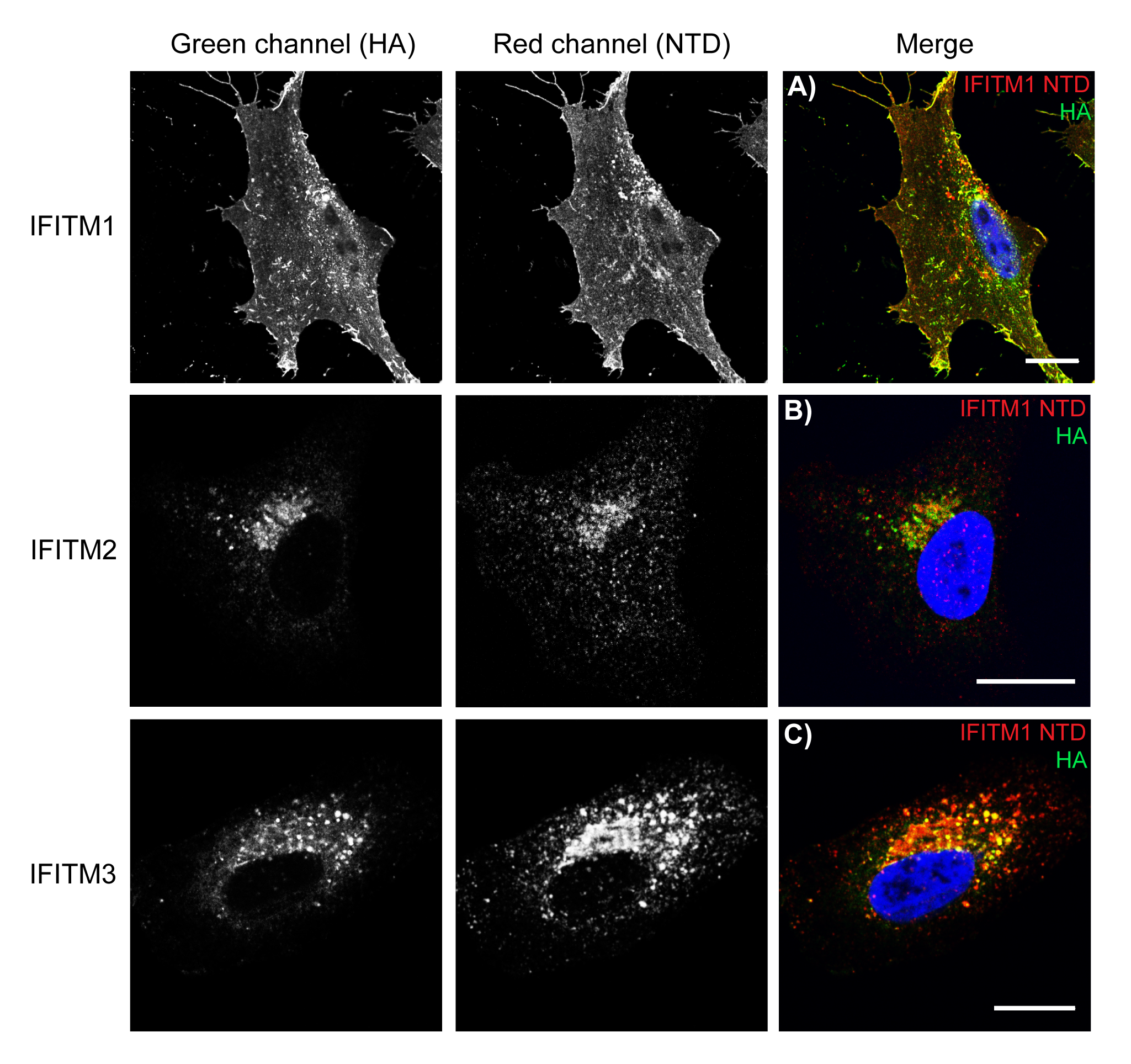

Supplement: Figure S6 — Co-staining with anti-IFITM1-NTD and anti-HA antibodies. Permeabilised IFITM1-HA (A), IFITM2-HA (B) and IFITM3-HA (C) expressing cells were stained with antibodies against the C-terminal HA-tag (green [Alexa-448]) and the NTD, using the anti-IFITM1-NTD antibody (red [Alexa-647]). Images are of single optical sections (0.25 µm thick) through the middle the cell. Scale bars represent 15 µm. (TIF) [file pone.0104341.s006.tif]
