# Supplementary material for: A Membrane Topology Model for Human Interferon Inducible Transmembrane Protein 1
Source: PLoS One. 2014 Aug 8;9(8):e104341. doi: 10.1371/journal.pone.0104341 (PMC4126714; doi:10.1371/journal.pone.0104341)
Supplement: Table S1 — Image analysis of anti-IFITM1-NTD antibody and anti-HA antibody co-labelling. Co-localisation analysis of multiple images, for each cell line, from three independent experiments. Pearson's R-value represents the correlation in intensity between the red (anti-IFITM1-NTD) and green (HA) channels. Mander's correlation coefficients, M1 and M2, represent the overlap of red, in pixels that are green, and the overlap of green, in pixels that are red, respectively. Relative areas of each colour were calculated as described in materials and methods. Error given is of the standard deviation. (DOCX) [file pone.0104341.s007.docx]

**Table S1**

| **Cell line** | **Number of cells imaged** | **Pearson’s R value** | **Mander’s M1** | **Mander’s M2** |
| --- | --- | --- | --- | --- |
| IFITM1 | 58 | 0.85 (±0.05) | 0.97 (±0.12) | 0.99 (±0.01) |
| IFITM2 | 53 | 0.60 (±0.1) | 0.83 (±0.17) | 0.82 (±0.12) |
| IFITM3 | 57 | 0.81 (±0.05) | 0.75 (±0.19) | 0.77 (±0.19) |

| **Cell line** | **Fields of view** | **Yellow relative area** | **Red relative area** | **Green relative area** |
| --- | --- | --- | --- | --- |
| IFITM1 | 14 | 0.70 (±0.18) | 0.15 (±0.13) | 0.15 (±0.13) |
| IFITM2 | 14 | 0.30 (±0.06) | 0.29 (±0.1) | 0.44 (±0.13) |
| IFITM3 | 15 | 0.25 (±0.08) | 0.55 (±0.14) | 0.20 (±0.1) |
